# EphB6 Receptor Modulates Micro RNA Profile of Breast Carcinoma Cells

**DOI:** 10.1371/journal.pone.0022484

**Published:** 2011-07-19

**Authors:** Lokesh Bhushan, Raj P. Kandpal

**Affiliations:** Department of Basic Medical Sciences, Western University of Health Sciences, Pomona, California, United States of America; University of Barcelona, Spain

## Abstract

Breast carcinoma cells have a specific pattern of expression for Eph receptors and ephrin ligands. EphB6 has previously been characterized as a signature molecule for invasive breast carcinoma cells. The transcription of EphB6 is silenced in breast carcinoma cells and its re-expression leads to decreased invasiveness of MDA-MB-231 cells. Such differences in phenotypes of native and EphB6 expressing MDA-MB-231 cells relate to an altered profile of micro RNAs. Comparative hybridization of total RNA to slides containing all known miRNAs by using locked nucleic acid (LNA) miRCURY platform yielded a significantly altered profile of miRNAs in MDA-MB-231 cells stably transfected with EphB6. After applying a threshold of change and a p-value of <0.001, the list of significantly altered miRNAs included miR-16, miR-23a, miR-24, miR-26a, miR-29a, miR-100, miRPlus-E1172 and miRPlus-E1258. The array-based changes were validated by real-time qPCR of miR-16, miR-23a, miR-24 and miR-100. Except miRPlus-E1172 and miRPlus-E1258, the remaining six miRNAs have been observed in a variety of cancers. The biological relevance of target mRNAs was predicted by using a common-target selection approach that allowed the identification of SMARCA5, SMARCC1, eIF2C2, eIF2C4, eIF4EBP2, FKABP5, FKBP1A, TRIB1, TRIB2, TRIB3, BMPR2, BMPR1A and BMPR1B as important targets of a subset of significantly altered miRNAs. Quantitative PCR revealed that the levels of SMARCC1, eIFC4, eIF4EB2, FKBP1a, FKBP5, TRIB1, TRIB3, BMPR1a and BMPR2 transcripts were significantly decreased in MDA-MB-231 cells transfected with EphB6. These observations confirm targeting of specific mRNAs by miR-100, miR-23a, miR-16 and miR-24, and suggest that the kinase-deficient EphB6 receptor is capable of initiating signal transduction from the cell surface to the nucleus resulting in the altered expression of a variety of genes involved in tumorigenesis and invasion. The alterations in miRNAs and their target mRNAs also suggest indirect involvement of EphB6 in PI3K/Akt/mTOR pathways.

## Introduction

Eph receptors belong to the largest class of receptor tyrosine kinases that are involved in a variety of processes such as hindbrain patterning, axon guidance and angiogenesis [1]. These molecules have also been implicated in several cancers, and considerable work has been done to understand their biological significance in tumorigenesis [2]. In addition to alterations in several Eph receptors and ephrin ligands, the expression of EphB6 receptor is transcriptionally silenced in invasive breast carcinoma cells [3]. The transcriptional silencing of EphB6 is attributed to the methylation of specific CpG dinucleotides in the gene promoter [4]. While the transcription of EphB6 gene is not detectable in MDA-MB-231 cells, transfection of an EphB6 expression construct significantly reduces *in vitro* invasiveness of these cells [5]. Yeast two-hybrid system has indicated possible interaction of EphB6 with a variety of intracellular proteins [6] that likely mediate phenotypes of EphB6-expressing cells. Comparative proteomic analysis of MDA-MB-231 cells have revealed that EphB6 either directly or indirectly affects the expression of a variety of proteins that are involved in metabolism, signal transduction, cytoskeleton and energy homeostasis [7].

The proteomic alterations observed in EphB6 expressing breast carcinoma cells [7] suggest that changes in the levels of some proteins may be direct effects of EphB6. However, a majority of the changes in protein levels appear to be indirect effects of EphB6. The underlying mechanisms for these changes could possibly include alterations in the abundance, stability and translatability of specific transcripts. Among a variety of regulatory molecules that control the expression of genes encoded in the human genome, miRNAs have emerged as a very important class of regulators. These 21 to 23 nucleotide long small molecules either enhance RNA degradation or prevent translation, and thus lead to changes in transcript levels as well as proteins [8]. It is predicted that greater than 2/3^rd^ of genes encoded in the human genome have sequences that may be targeted by a variety of miRNAs. The disease relevance of miRNAs is evident from the altered levels of these molecules in various cancers [9], [10]. The introduction of a specific miRNA construct in tumor cells has been shown to suppress tumor phenotypes [11], thus attributing an important regulatory role to miRNAs. Given the large numbers of predicted targets for specific miRNAs, a single miRNA can influence the abundance of a large number of mRNAs and proteins [12].

Based on the altered proteomic profile of EphB6-transfected MDA-MB-231 cells [7] it is hypothesized that EphB6 transfection likely influences protein profile by modulating the abundance of miRNA complement of the cell. To address this possibility and establish a relationship between cellular phenotype and miRNAs, an array-based hybridization strategy was carried out using the locked nucleic acid (LNA) miRCURY platform. We present here the relationship between miRNAs and EphB6 expression status of MDA-MB-231 cells and the biological relevance of these changes for cancer cell phenotypes.

## Results

### Quality of RNA used for hybridizations

The yield of total RNA from different samples, as determined by their absorbance at 260 nm, varied between 50 and 70 µg, and the ratios of absorbance at 260 nm and 280 nm for various samples ranged between 2.01 and 2.05. The ratios of 28S and 18S rRNAs in total RNA preparations made from vector-transfected and EphB6-transfected clones varied between 1.8 and 1.9, thus confirming that the samples have not undergone any detectable degradation. The electropherogram revealed RNA integrity numbers for various samples ranging between 9.7 and 9.8. These results indicated the RNAs to be of high quality and well-suited for performing miRNA profiles.

### EphB6 transfection leads to changes in miRNA profiles

The hybridization of total RNA isolated from vector-transfected and EphB6-transfected MDA-MB-231 cells to miRNA arrays revealed detectable expression of a large number of miRNAs, as indicated by significantly higher signal intensities at spots corresponding to these miRNAs on the slides as compared to the background. These signal intensities were subsequently compared to determine a subset of miRNAs that were differentially expressed in EphB6-transfected MDA-MB-231 cells. The heatmap and alterations in the levels of specific subsets of miRNAs at different *p*-values are described in the following sections.

**Figure 1:**
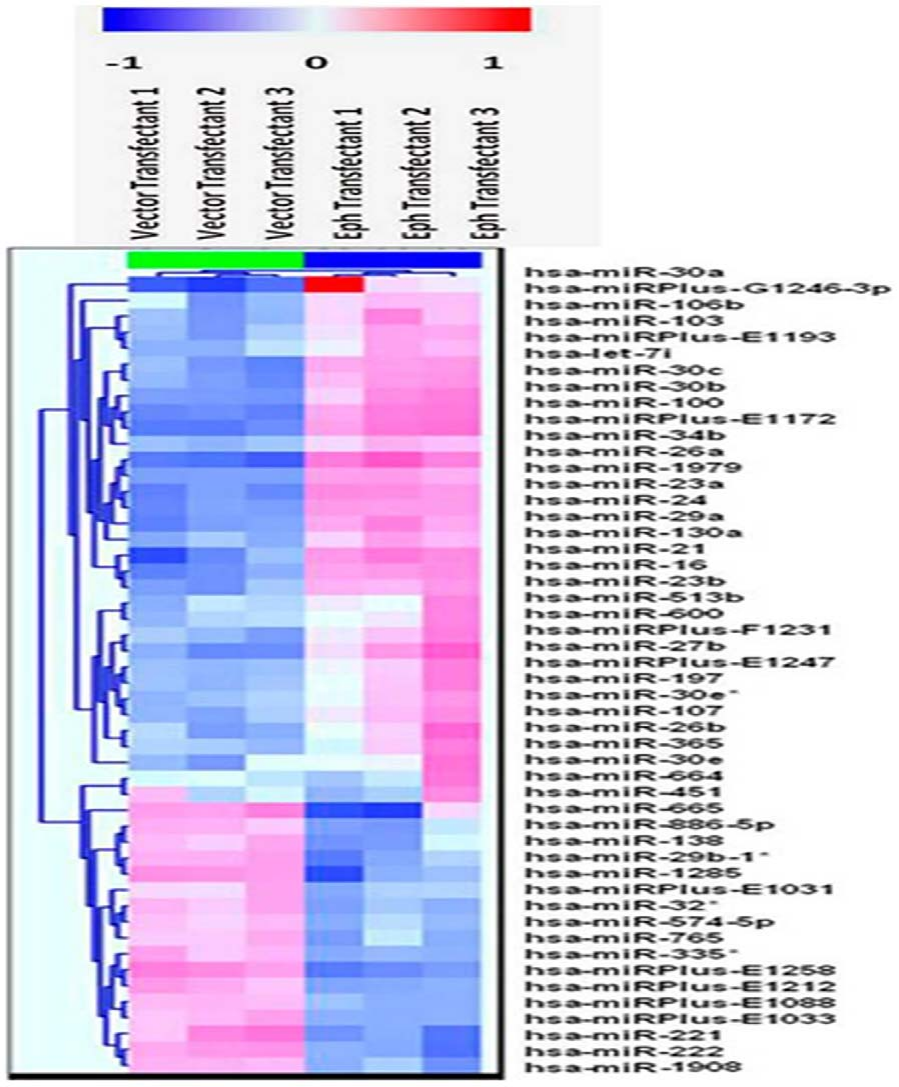
Heatmap of altered miRNAs in EphB6-transfected MDA-MB-231 cells. A two-way hierarchical clustering was performed to generate the heatmap. Each row represents a miRNA and each column represents a sample, and the miRNA clustering tree is shown on the left. Red color represents an expression level above mean and blue color represents expression level lower than the mean. Top 50 miRNAs altered, based on variation across replicates, with a p-value of <0.001 for three vector-transfectants and three EphB6 transfectants are shown.

Two-way hierarchical clustering of the data allowed the construction of the heatmap shown in Figure 1. Since a common reference pool was used for hybridization, comparisons of samples were made with the common reference pool. The differences in intensities at individual spots on the array were recorded as log_2_(Hy3/Hy5) values, where Hy3 is sample fluorescence and Hy5 is fluorescence contributed by the common reference pool [13]. Top 50 candidate miRNAs, based on variations across samples, are represented in the heatmap. It is evident from Figure 1 that 33 miRNAs were upregulated and the levels of 17 miRNAs had decreased in EphB6-transfected cells as compared to the empty vector-transfected MDA-MB-231 controls. These miRNAs were subsequently categorized in two smaller subsets based on *p*-values as described below.

The data were analyzed by applying a two-tailed t-test between the two sample groups of RNAs isolated from vector-transfected and EphB6-transfected MDA-MB-231 cells, respectively. The miRNAs that showed alterations with a *p*-value of less than 0.001 are shown in Table 1. There are 34 miRNAs that satisfied the *p*-value cut off. Among the significantly altered miRNAs, 20 miRNAs were up-regulated while 14 miRNAS are down-regulated.

**Table 1:** Altered miRNAs in EphB6-transfected MDA-MB-231 cells.

Upregulated miRs@	Downregulated miRs@
hsa-miR-26a	hsa-miRPlus-E1258
hsa-miRPlus-E1172	hsa-miRPlus-E1088
hsa-miR-100	hsa-miRPlus-E1033
hsa-miR-23a	hsa-miR-335*
hsa-miR-16	hsa-miRPlus-E1212
hsa-miR-29a	hsa-miRPlus-E1117
hsa-miR-24	hsa-miR-32*
hsa-miR-1979	hsa-miR-720
hsa-let-7i	hsa-miR-222
hsa-miR-30d	hsa-miR-1908
hsa-miR-30a	hsa-miR-221
hsa-miR-191	hsa-miR-1285
hsa-miR-30c	hsa-miR-183
hsa-miR-34b	hsa-miR-665
hsa-miR-130a	
hsa-miR-125b	
hsa-miR-23b	
hsa-miR-30b	
hsa-miR-21	
hsa-miR-27b	

@ Array hybridizations were performed with RNA isolated from three independent clones of vector-transfected and EphB6-transfected DNA as described in the Methods' section. The altered RNA at a p-value of <0.001 are presented here.

**Figure 2:**
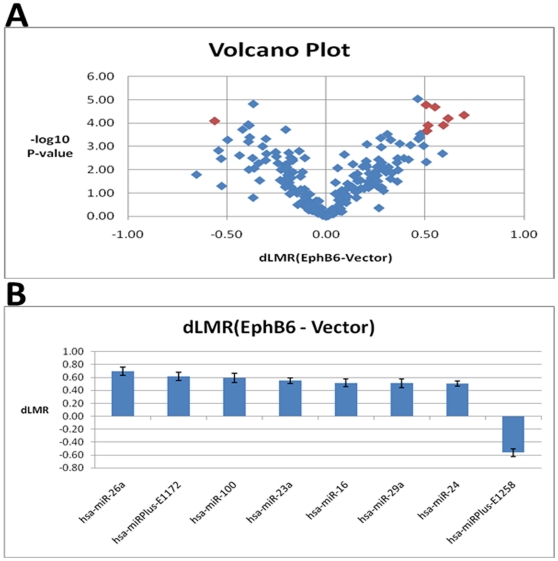
Significantly altered miRNAs in EphB6 transfected MDA-MB-231 cells. A. Volcano plot indicating miRNAs with dLMR values greater than 0.5 or less than −0.5 at a p-value of less than 0.001. The symbols in red color represent the miRNAs meeting the cut-off values. B. Variations in the values of delta LogMeadianRatio (dLMR) for selected miRNAs are indicated by standard deviations among three replicates.

**Figure 3:**
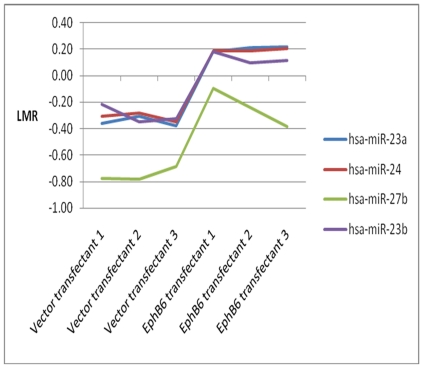
Coordinated changes in the levels of members of specific miRNA families. The levels of expression for two miRNA clusters in three vector-transfectants and three EphB6-transfectants are shown.

The array data were filtered by applying absolute Δ LogMedianRatios (dLMR), and the results are shown as a Volcano plot (Figure 2A). These analyses revealed significant alterations in 8 miRNAs comprising of miR-26a, miRPlus-E1172, miR-100, miR-23a, miR-16, miR-29a, miR-24 and miRPlus-E1258 that were altered with a *p*-value of less than 0.001 and dLMR either greater than 0.5 or less than −0.5. Among these miRNAs, seven were up-regulated and miRPlus-1258 was significantly down-regulated in EphB6 transfected cells. The differences in the above miRNAs were further confirmed by plotting the dLMR with standard deviations. The standard deviations for triplicate measurements of vector-transfected and EphB6-transfected samples were well within the acceptable range (Figure 2B). A coordinated expression pattern was observed for members of two polycistronic miRNA clusters containing miR-23a, miR-23b, miR-24 and miR-27. The levels of miR-23a, miR-23b and miR-24 were comparable in all vector-transfected MDA-MB-231 clones. However, their levels in EphB6-transfected clones were significantly higher than the vector-transfected clones. Similar changes were observed in the levels of miR-27 between vector-transfected and EphB6-transfected clones (Figure 3).

### Real-time qPCR validation of specific miRNAs

**Figure 4:**
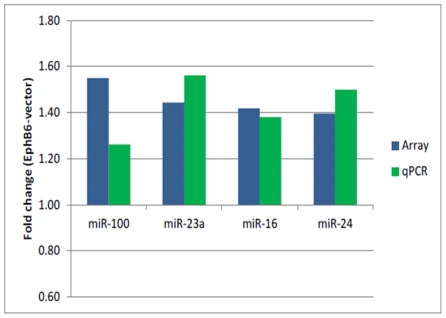
Real-time qPCR for significantly altered miRNAs. Total RNA from two clones of vector-transfected and EphB6-transfected cells were amplified with specific primers as described in the Methods' section. Each reaction was carried out in triplicate. The fold-changes in expression obtained from array hybridization and qPCR are shown.

Of the eight significantly altered miRNAs, miR-100, miR-23a, miR-16 and miR-24 were validated by qPCR. The comparison of results from array hybridization and qPCR is shown in Figure 4. The qPCR fold-changes in the levels of these miRNAs in EphB6-transfected cells ranged between 1.3 and 1.55. It warrants mention that array results indicated changes of 1.4 to 1.55 fold in the levels of these miRNAs in EphB6-transfected cells. Quantitation by qPCR revealed that the levels of miR-16 and miR-24 in EphB6-transfected cells were upregulated by 1.39 and 1.45 fold, respectively. The levels of these two miRNAs by array hybridization were found to be 1.42 and 1.41-fold higher in EphB6-transfected cells as compared to the vector-transfected cells. While array-hybridization showed 1.55 and 1.46-fold elevations in the levels of miR-100 and miR-23a, qPCR revealed 1.3 and 1.55-fold alterations in their levels. These results clearly demonstrate that the miRNA abundance differences observed by array hybridization are comparable to qPCR results.

### Target mRNAs for miRNAs and prediction of biologically relevant mRNAs by using a common target prediction approach

Given the numerous mRNAs targeted by a single miRNA and alterations in a large number of miRNAs in a specific condition, it becomes difficult to identify biologically relevant targets. A common target prediction approach was used to focus on the relevancy of miRNAs. Out of the eight miRNAs that were significantly altered, four miRNAs were selected and their predicted target mRNA lists compared (http://www.targetscan.org/). As shown in Table 2, miR-99ab/100 family, miR-23a family, miR-16 family and miR-24 family have 40, 838, 968 and 438 mRNA targets, respectively. Of the 40 target mRNAs predicted for miR-99ab/100 family, there were only 9 targets for miR-100 (Table 2). These 9 targets were compared with the lists of predicted targets for miR-23a, miR-16 and miR-24. The comparisons revealed that five miR-100 targets, namely, SMARCC1, eIF4EBP2, FKB5, TRIB1 and BMPR1B, were represented in the target lists of other miRNAs. In some cases, the targets included either isoforms of these mRNAs or mRNAs involved in related functions. It warrants mention that these genes represent a small fraction of a combined set of mRNAs targeted by four miRNAs. Since the target lists for miRNAs include a large number of mRNAs, we believe that the combinatorial scheme described above may allow prioritization of target mRNAs for validation.

**Table 2:** Common or functionally related mRNAs targeted by four miRNAs*.

miR-100	miR-23a	miR-16	miR-24
Target mRNAs: 9	Target mRNAs for miR-23a/b: 838	Target mRNAs for miR-16 family: 968	Target mRNAs for miR-24 family: 438
HS3ST2			
SMARCA5	SMARCC1		
EPDR1			
ZZEF1			
EIF2C2	EIF4EB2	EIF2C4	
FRAP1			
FKBP5	FKBP5	FKBP1A	
TRIB2	TRIB1		TRIB3
BMPR2	BMPR1B	BMPR1A	BMPR2, BMPR1B

*The *in silico* predictions of target mRNAs were obtained from TargetScan and miR-100 targets were searched against the lists of targets for miR-23a, miR-16 and miR-24 as described in the Methods' section.

### Validation of target mRNAs

**Figure 5:**
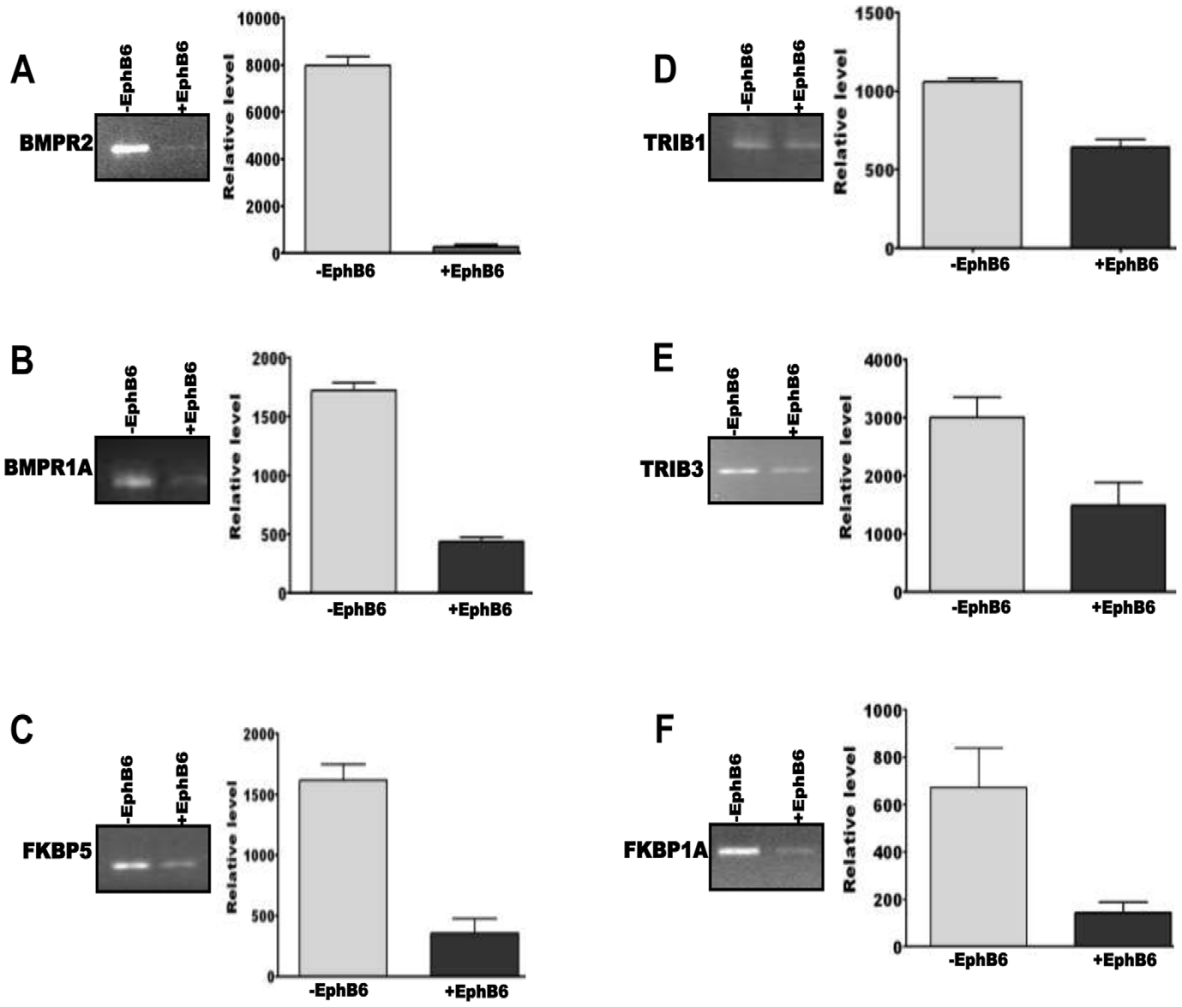
Amplification of mRNAs targeted by various miRNAs. Each panel shows a representative gel picture for the transcript levels in empty vector-transfected (left lane) and EphB6-transfected (right lane) MDA-MB-231 cells. The bar diagram on the right shows quantification of three gels for the levels of transcripts. The Y-axis represents arbitrary units of intensity. Empty vector-transfected and EphB6-transfected MDA-MB-231 cells are designated as −Eph and +Eph. The transcript designations are indicated in each panel.

**Figure 6:**
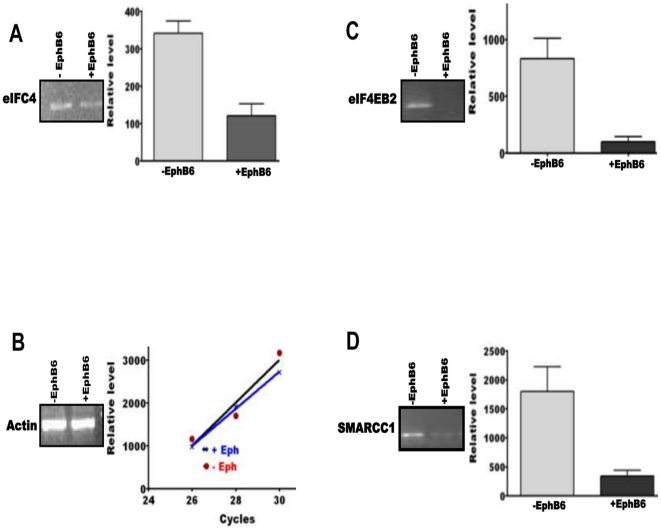
Amplification of mRNAs targeted by various miRNAs. Each panel shows a representative gel picture for the transcript levels in empty vector-transfected (left lane) and EphB6-transfected (right lane) MDA-MB-231 cells. The bar diagram on the right shows quantification of three gels for the levels of transcripts. The Y-axis represents arbitrary units of intensity. Empty vector-transfected and EphB6-transfected MDA-MB-231 cells are designated as −Eph and +Eph. The transcript designations are indicated in each panel. The graph shows linear and comparable amplification of actin transcript in RNA isolated from empty vector-transfected and EphB6-transfected MDA-MB-231 cells.

We selected SMARCC1, eIF2C4, eIF4EBP2, FKABP5, FKBP1A, TRIB1, TRIB3, BMPR2 and BMPR1A for validation by PCR. As shown in Figures 5 and 6, the levels of these transcripts were significantly down-regulated in MDA-MB-231 cells transfected with EphB6. Noteworthy among these targets were BMPR2 and eIF4EB2. The mRNAs for BMPR2 and eIF4EB2 were barely detectable in EphB6 transfected cells. The levels of SMARCC1, eIF2C4, FKABP5, FKBP1A, TRIB1, TRIB3 and BMPR1A in the transfected cells were between 20% and 30% of the native cells. These results validate the rationale for the selection approach, and clearly demonstrate the low abundance of a target mRNA for which the relevant miRNA is up-regulated in the cell. These results clearly demonstrate an inverse relationship between the levels of miRNAs and their mRNA targets.

## Discussion

Micro RNA profiles have been studied for a number of cancers, and alterations in a subset of miRNAs have been characterized. Given the regulatory role played by miRNAs, it is predicted that these regulatory molecules serve an important function in modulating a variety of processes including tumor initiation, growth and metastasis. The transfection of EphB6 into MDA-MB-231 cells has been shown to decrease invasiveness and ability to form foci in soft agar [5]. These phenotypic changes were subsequently correlated to altered proteomic profile of EphB6-transfected MDA-MB-231 cells [7]. Such phenotypic changes and altered proteomic profiles suggested that EphB6 is capable of triggering global molecular changes, and such a claim is supported by the alterations in miRNA profiles of EphB6-transfectants described here. Coordinated changes in expression of a family of miRNAs observed in EphB6-transfected cells indicate organization and regulation of miRNAs based on their functional significance. The cut-off values applied for data analysis have allowed the selection of fewer miRNAs as relevant for the invasive phenotype. Four of these miRNAs were confirmed by real-time qPCR, which validated the results of the array platform. Six out of the top eight miRNAs are involved in a variety of processes and also associated with human cancers. It has been shown that miR-26a acts via EZH2, an oncogene markedly elevated in breast cancer cells [14], and affects growth and tumorigenesis [15]. The delivery of miR-26a in cancer cells inhibits proliferation [11]. MiR-100 overexpression has been shown to inhibit mTOR pathway [16]. MiR-23a, which regulates osteoblast differentiation [17] and is under the control of Myc, is known to be upregulated in breast cancer [18]. MiR-16, an inhibitor of Wip1, renders MCF7 cells sensitive to doxorubicin [19]. MiR-29a antagonizes invasiveness of lung carcinoma cells [20], and it also leads to epithelial mesenchymal transition (EMT) and metastasis by suppressing the expression of tristetrapolin [21]. MiR-24 is known to target VEGFR1 (FLT1) and also MMP14 [10], and its under-expression has been observed in 4-OH tamoxifen-resistant MCF7 cells [22].

A target-based approach is discussed below to extract biological relevance of changes in abundance of miR-16, miR-23a, miR-24 and miR-100. Although miR-99ab/100 family has 40 targets, only 9 of these targets are assigned to miR-100 in TargetScan (http://www.targetscan.org/). Thus the numbers of target mRNAs for miR-100, miR-23a/b, miR-16 family and miR-24 family range between 9 and 968. Nine mRNA targets of miR-100 were searched against the targets of miR-16 family, miR-23a/b and miR-24 family. FKBP5 is a common target of miR-100 and miR-23a, while SMARCC1 and TRIB1 share functional similarity with the targets of miR-100. Furthermore, eIF2C4, FKBP1A, TRIB3, BMPR2 and BMPR1A are other targets that are common between two of the four miRNAs. The validation of this selection approach is evident from the observations that each one of the target mRNAs was down-regulated in cells that over-express its regulator miRNA. Based on these results, we suggest that the common-target selection approach described above may help identify the most likely candidates from the list of *in silico* predicted target mRNAs. As discussed below, changes in the abundance of these transcripts have been shown in a variety of human cancers. SMARCC1 is altered in prostate and colorectal cancer [23] and SMARCA5 is known to be dysregulated in leukemia [24] and up-regulated in human breast tumors [25]. The gene for eIF2C2 maps to a region of gain in breast cancer located at 8q24 [26] and belongs to the argonaute (AGO) family of genes. Ago2 or eIF2C2 is a core component of the RNA-induced silencing complex (RISC). The transcripts for eIF2C2 and eIF2C4 and their corresponding miRNAs are known to be coordinately altered in breast cancer [27]. A negative regulator of translation, eIF4EBP2, has been shown to be altered in breast cancer cells upon inhibition of proteasome [28]. FKBP5 is down-regulated in breast cancer [29], and its abundance in cancer cells renders them sensitive or resistant to gemcitabine and AraC. While higher levels of FKBP5 confer sensitivity, low amounts of FKBP5 are associated with resistance [29]. FKBP12 or FKBP1A has been shown to inhibit TGFβ type 1 receptor [30], and its overexpression has been reported in astrocytoma [31]. Mammalian homologues of the Tribles (Trib) family of proteins mediate proteasome-dependent degradation. These proteins have also been implicated in cancer phenotypes. Trib1 is involved in the etiology of AML [32] as well as ovarian cancer [33]. Trib2 has been shown to be a repressor of FOXO in malignant melanoma [34], and Trib3 expression is altered in colorectal cancer [35]. Although molecular basis of these changes has not been well-understood, we report here some involvement of EphB6 in these processes via alterations in the levels of various miRNAs.

From these data it appears that EphB6 effects may be operative at two levels. First, its interactions with other proteins may activate signaling molecules that are responsible for the altered transcription of their target genes. Second, the products of these target genes are affecting the transcription of another set of genes. Given the numerous changes in proteomic profile of EphB6-transfected cells [7], it is difficult to conclude which miRNAs represent the primary effects of EphB6. It has been previously shown that several enzymes/factors involved in general metabolism are altered in EphB6-transfected cells [7]. The alterations in miR-23a that targets eIF4EBP2 suggest changes in the rates of protein synthesis and these changes appear to correlate with the altered proteomic profiles observed in EphB6-transfected cells [7]. Among the numerous targets of several miRNAs affected in an EphB6-dependent manner, interaction of Trib with FOXO assumes cancer related significance. FOXO proteins, which belong to the forkhead family of transcription factors, are regulated by the insulin/PI3K/Akt signaling pathway. These proteins control the transcription of a variety of genes involved in cell cycle arrest, cell death, DNA repair and energy homeostasis [36]. Since the effects of EphB6 signaling on cell cycle, cell death and DNA repair have not been studied adequately, the changes in enzymes involved in bioenergetics pathways in EphB6-transfected MDA-MB-231 cells [7] suggest that EphB6 may alter energy homeostasis. MMP14, a target of miR-24, also corroborates our earlier studies indicating the down-regulation of MMP7 and MMP19 in EphB6-transfected MDA-MB-231 cells [5].

The alterations in the level of miR-100 assume significance in the context of PI3K/Akt/mTOR pathway proteins. It is well-established that PI3K/Akt/mTOR pathways are activated in a variety of cancers, and these pathways are being explored as targets for therapeutic intervention of cancers [37]. PI3K/Akt pathway is known to regulate FOXO, which is repressed by Trib2, a target of miR-100. Thus EphB6 may independently alter the abundance of target proteins of PI3K/Akt pathway. Secondly, the linkage between miR-100 overexpression and inhibition of mTOR pathway [16] supports invasiveness-suppressor effects of EphB6 via mTOR pathway proteins. Similar interpretation may be forwarded to establish a link between miR-16, EphB6 and the change in the invasiveness of MDA-MB-231 cells. Mir-16 is known to be expressed in breast tumor cells, and its elevated levels in circulation have been related to better prognosis of myelodysplastic syndromes [38]. We speculate that the decrease in invasiveness of EphB6-expressing breast carcinoma cells may be mediated by miR-16 in a concentration-dependent manner. The biological significance of these changes may be explained by associating specific consequences of EphB6 protein with its abundance as well as the levels of other Eph receptors in breast carcinoma cells. Our results also suggest that EphB6 may be exploited as a target for therapeutic intervention of breast cancer regardless of the specificity of its downstream effects.

The results presented here describe a testable strategy for confirming biologically relevant targets of various miRNAs. Such strategies become important because individual miRNAs have hundreds of target mRNAs, and a large number of miRNAs are altered when cellular phenotypes change during the natural course of a disease or following transfection of specific cDNAs. Given that the specific miRNAs altered in EphB6-transfected cells have been shown in a variety of cancers and cellular processes, the strategy of focusing on a set of common mRNAs targeted by two or more miRNAs assumes significance for their implications on a given phenotype and underlying mechanisms. Although our data do not permit us to assign a specific miRNA to EphB6-mediated pathways, we suggest that direct and/or indirect downstream effects of EphB6 signaling may include a variety of miRNAs. These miRNAs may alter the abundance of proteins involved in cellular metabolism as well as phenotypes that are hallmarks of breast carcinoma cells. Based on the results presented here, it is concluded that miR-100, miR-23a, miR-16 and miR-24 may act synergistically to bring about the invasion suppressive phenotype observed in EphB6-transfected MDA-MB-231 cells.

## Materials and Methods

### Cell lines and transfections

Breast carcinoma cell line MDA-MB-231 (ATCC, Manassas, VA, USA) was cultured at 37°C in the presence of 7% CO_2_ in a medium containing DMEM (Gibco) with 10% fetal bovine serum (Hyclone, Logan, UT, USA), 2.0 mM L-glutamine (Gibco), 1.0 mM sodium pyruvate (Gibco), 25 U/ml penicillin (Gibco) and 25 mg/ml streptomycin (Gibco) [5]. The cells in logarithmic phase were transfected with either an empty vector or an expression construct of EphB6 in pCDNA3.1 using Lipofectamine. The transfected cells were grown in the presence of 500 µg/ml G418 for 2–4 weeks and three surviving clones each of vector transfected and EphB6 transfected cells, respectively, were isolated, expanded and stored [5].

### RNA isolation and characterization

The vector- or EphB6-transfected clones were grown as described above. RNA was isolated from 10 cm tissue culture dishes when cells were 85–95% confluent using TRIZOL reagent (Invitrogen) as per the recommended protocol. Briefly, 1 ml of reagent was used per 10 cm dish followed by the addition of 200 µl chloroform, and the aqueous phase was separated by centrifugation in a microfuge. RNA was precipitated by adding 250 µl isopropanol and isolated as a pellet by centrifugation. The pellet was then washed sequentially with 80% and 100% ethanol, and air-dried. RNA was dissolved in DEPC-treated H_2_O, and stored in aliquots at −80°C.

The concentration of RNA was determined by absorbance measurements at 260 nm in a nanodrop spectrophotometer. Contaminating proteins and organic compounds such as phenol in the RNA preparation were checked by determining absorbance ratios 260 nm/280 nm and 260 nm/230 nm, respectively. The quality of RNA was characterized by electrophoresis in Agilent Bioanalyzer 2100. The electropherogram allowed the quantitation of 28S rRNA and 18S rRNA as well as the miRNA fraction separated at 25–30 sec. The appearance of smear or multiple small peaks in the range between 25 and 50 sec are indicative of RNA degradation, and these factors were used to calculate the RNA integrity number [39].

### RNA labeling and hybridization to miRNA slides

A common reference sample was made by pooling an aliquot of each of thxe samples in the experiment. RNA from individual samples and the common reference pool were labeled with Hy3™ and Hy5™ fluorescent label, respectively, using the miRCURY™ LNA Array power labeling kit (Exiqon, Denmark) following the procedure described by the manufacturer. The Hy3™-labeled samples and a Hy5™-labeled reference RNA sample were mixed pair-wise and hybridized to the miRCURY™ LNA Array version 5^th^ Generation (Exiqon, Denmark), which contains capture probes targeting all miRNAs for human, mouse or rat registered in the miRBASE version 15.0 at the Sanger Institute. The hybridization was carried out as recommended by the vendor. After hybridization the microarray slides were scanned and the images analyzed. The analysis of the scanned slides indicated successful labeling of RNAs as evidenced by the signal intensities of all capture probes for the control spike-in oligonucleotides. The correlation coefficients between slides for the spike-in controls for Hy3 as well as Hy5 channels were near unity. The quantified signals were background corrected [40] and normalized using the global ‘locally weighted scatterplot smoothing’ (LOWESS) regression algorithm. These normalizations were performed to minimize differences between the colors in an intensity-dependent manner.

### Real-time qPCR for validating selected miRNAs

Total RNA (10 ng) was reverse transcribed in triplicates in 10 µl reactions using the miRCURY LNA™ Universal RT miRNA PCR, polyadenylation and cDNA synthesis kit (Exiqon), and the cDNA was diluted 100-fold. Each PCR was carried out in a total volume of 10 µl by using 4 µl of the diluted cDNA according to the miRCURY LNA™ Universal RT miRNA PCR protocol. The amplification patterns were used to evaluate melting curves, and Cp (crossing point) values for each miRNA were determined by LightCycler 480 software release 1.5.0. The amplification efficiencies were calculated by using LinRegPCR [41], and the average efficiency of each reaction was used to correct the Cp values.

A mixture of RNAs from 20 different tissues was used as a positive control. Three negative controls consisted of reactions containing no RNA in the reverse transcription step, no enzyme in the reverse transcription step and no cDNA in the qPCR step, respectively. Three miRNAs (miR-let-7a, miR-423-3p and miR-22) were used as reference. These miRNAs were found to have the best stability values, as calculated by SLqPCR-R package, and used to normalize the quantified signal. Based on the geomean Cp of the reference miRNAs, data were normalized on a well-to-well basis and ΔCp values were calculated.

### Validation of target mRNAs by PCR

A set of target mRNAs predicted by TargetScan (http://www.targetscan.org/) was selected for validation. Primers specific to selected mRNAs (Table 3) were designed and semiquantitative PCR was performed by using equal amounts of templates corresponding to empty vector-transfected and EphB6-transfected MDA-MB-231 cells. Total RNA (1.8 µg) was reverse transcribed by using SuperScript II reverse transcription kit (Invitrogen) as recommended by the manufacturer. The cDNA was diluted 1∶20 and an aliquot was amplified for 30–40 cycles by using annealing temperatures specific to various primers. Template concentration was normalized by amplifying actin transcript. The sequences of primers, annealing temperatures and amplification cycles are presented in Table 3.

**Table 3:** Sequences of primers used for PCR.

Gene	Gene Name	Location	Forward Primer	Reverse Primer	Temp (°C)	Cycles	Size (bp)
SMARCC1	SWI/SNF related, matrix associated, actin dependent regulator of chromatin, subfamily c, member 1	3p21.31	CTGGCTGGGCTGGTGGTGCAGC	TCTTCCCCAAGGTCAACTGATC	55	40	1091
FKBP5	FK506 binding protein 5	6p21.31	GAGTGGGGAATGGTGAGGAAACG	CCCCTTCTGAAGTCTTCTTGCCC	55	30	1194
FKBP1A	FK506 binding protein 1A	20p13	GGCGCACCTTCCCCAAGCGCGG	GGATGATGCCTGGGTGCCCAGT	65	30	240
TRIB1	Tribbles homolog 1	8q24.13	CCCATTAAACACTACCAGGAC	TCAGGTCCCCCAGCACGATGGCT	55	40	260
TRIB3	Tribbles homolog 3	20p13-p12.2	CCCCGTCCAGGAAGCCCTGGCC	CTCTCACGGTCAGCGAAGAC	55	40	288
BMPR1A	Bone morphogenetic protein receptor, type IA	10q22.3	TGCTTCATGGCACTGGGATG	GCAGGTGGCACAGACCACAGGC	65	35	973
BMPR2	Bone morphogenetic protein receptor, type II	2q33-q34	TAGACTGGTGCGCCCAGGGG	AGCAGACAGGGGTTGGCCCA	65	30	950
eIF2C4	Eukaryotic translation initiation factor 2C, 4	1p34.3	CGGACCTCCGGCTAGCCTGT	CCAGACCTCCCTGCCCCCTC	65	30	550
eIF4EBP2	Eukaryotic translation initiation factor 4E binding protein 2	10q21-q22	GAACTCGAATCATTTATGACAG	CAACTGCATGTTTCCTGTCGTG	55	40	189
ACTB	Actin-beta	7p22	CTGACTGACTACCTCATGAAG	ATCCACATCTGCTGGAAGGTG	55	20–30	517

Equal amounts of cDNA made from total RNA of empty vector-transfected and EphB6-transfected MDA-MB-231 cells were amplified and an aliquot of the amplified DNA was electrophoresed in an agarose gel. Each amplification was performed in triplicate, and the band intensity quantified by using Quantity One 1-D Analysis Software Version 4.6.5 (BIORAD), and the results were presented as mean ± standard deviation.
